# Yellow Fever

**Published:** 1867-10

**Authors:** 


***Yellow Fever.***

The history of the epidemic which is at present devastating portions of the Gulf States will tend to draw still more strongly official and popular attention to the necessity of improved sanitary methods, and persistence in well-doing. The key-note to the whole chorus, is: "*All malignant epidemics are preventible,*" cholera, yellow fever, or whatsoever the nomenclature.

Put away *concurrent* causes of diseases, and no single exciting cause, however great its supposed virulence, will be competent to beget wide-spread pestilence.

